# Development of a youth version of the Here and Now Aboriginal Assessment (HANAA-Y) tool

**DOI:** 10.1192/j.eurpsy.2023.378

**Published:** 2023-07-19

**Authors:** A. Janca, Z. Lyons

**Affiliations:** Psychiatry, University of Western Australia, Claremont, Australia

## Abstract

**Introduction:**

Assessment of social and emotional wellbeing (SEWB) of Aboriginal people is challenging. A The culturally appropriate screening instrument for SEWB in Aboriginal adults entitled Here and Now Aboriginal Assessment (HANAA), has been developed and evaluated. The HANAA explores ten key domains and adopts a yarning process to initiate a semi-structured interview that covers each domain. This is recorded in narrative form and each domain rated as ‘problem’ or ‘no problem’ and a ‘recommended action’ is determined. The HANAA is widely used by Aboriginal mental health service providers around Australia.

**Objectives:**

There have been multiple requests by service providers for a similar instrument to be developed for young Aboriginal people. This study aims to develop a youth version of the instrument abbreviated as HANAA-Y.

**Methods:**

A Working Group comprised of Aboriginal and non-Aboriginal psychiatrists and mental health professionals with expertise of SEWB in Aboriginal youth was established and a draft version of the HANAA-Y has been produced. Evaluation od cultural applicability, reliability and validity of HANAA-Y is underway in metropolitan, rural and remote locations across Australia.

**Results:**

The original HANAA structure, yarning style, and rating has been retained. However, new domains and probe words relevant to young people have been selected. The new domains are as follows: somatic complaints; emotional issues; suicide risk and self-harm; alcohol and drug use; cognition and activity; behavioural and legal issues; strange thoughts and unusual experiences; functioning; stressful life events; and resilience and healing. The HANAA-Y administration guidelines have also been amended to be of relevance to Aboriginal youth.

**Conclusions:**

It is expected that HANAA-Y will be a culturally appropriate and useful instrument which can be used by a range of service providers with differing levels of mental health training to screen for SEWB among young Aboriginal people.

**Disclosure of Interest:**

None Declared

